# Deformation-resembling microstructure created by fluid-mediated dissolution–precipitation reactions

**DOI:** 10.1038/ncomms14032

**Published:** 2017-01-27

**Authors:** Liene Spruzeniece, Sandra Piazolo, Helen E. Maynard-Casely

**Affiliations:** 1 ARC Centre of Excellence for Core to Crust Fluid Systems/GEMOC, Department of Earth and Planetary Sciences, Macquarie University, Sydney, New South Wales 2109, Australia; 2 Australian Centre for Neutron Scattering, Australian Nuclear Science and Technology Organisation, Locked Bag 2001, Kirawee DC, New South Wales 2232, Australia

## Abstract

Deformation microstructures are widely used for reconstructing tectono-metamorphic events recorded in rocks. In crustal settings deformation is often accompanied and/or succeeded by fluid infiltration and dissolution–precipitation reactions. However, the microstructural consequences of dissolution–precipitation in minerals have not been investigated experimentally. Here we conducted experiments where KBr crystals were reacted with a saturated KCl-H_2_O fluid. The results show that reaction products, formed in the absence of deformation, inherit the general crystallographic orientation from their parents, but also display a development of new microstructures that are typical in deformed minerals, such as apparent bending of crystal lattices and new subgrain domains, separated by low-angle and, in some cases, high-angle boundaries. Our work suggests that fluid-mediated dissolution–precipitation reactions can lead to a development of potentially misleading microstructures. We propose a set of criteria that may help in distinguishing such microstructures from the ones that are created by crystal-plastic deformation.

## Abstract (web summary)

Microstructural features of deformed rocks are used to reveal deformation stresses and temperatures. Here, the authors conduct experiments showing that misleading microstructures form during fluid-mediated mineral reactions under static conditions, and propose new criteria for microstructure identification.

During the past 20 years, major efforts have been made to develop quantitative methods for obtaining information on deformation conditions and large-scale tectonic processes from a rock microstructure. The microstructural features of deformed rocks, such as the size of recrystallized grains/subgrains, the crystallographic preferred orientation patterns, intracrystalline lattice misorientations and grain/subgrain boundary geometries, have been used to determine the magnitude of differential stresses^1^,^2^, deformation temperatures^3^, mode of deformation in terms of vorticity and stress axes^4^ and atomic scale deformation mechanisms^5^,^6^.

However, recent experimental studies demonstrate that if a mineral undergoes chemical alteration by interaction with a reactive fluid, the general crystallographic features of the parent grains can be transferred to the reaction products^7^. Such inherited crystallographic preferred orientation patterns are suggested as a potential cause for the anisotropy in mantle serpentinites^8^, mantle wedge peridotites^9^, post-perovskite in the lower mantle^10^ and a variety of metasomatized crustal rocks^11^,^12^. Furthermore, it has also been shown by Raufaste *et al*.^13^ and occasionally indicated by field studies^14^,^15^,^16^ that products of such fluid-mediated reactions exhibit high amount of structural defects in their crystal lattices. These findings pose important questions on the characteristics of the microstructures resulting from fluid-mediated mineral reactions. What is the angular and spatial scale of inherited features in such microstructures? Can crystal nucleation and growth processes produce microstructures that mimic those resulting from crystal-plastic deformation processes? Failing to correctly interpret the origin of such reaction-induced microstructures will result in errors in the reconstructions of the geological history of an area. Conversely, correct interpretation will markedly aid recognition of metasomatic events important not only for the fundamental understanding of a rock but also its economic potential.

Here we present the first experimental study, where the microstructural development during fluid–rock interaction is explored with high-resolution routine characterization techniques (electron backscatter diffraction (EBSD)) that are now increasingly used by structural and metamorphic geologists. The results provide unique insights into the microstructural development of minerals during their interaction with a fluid and have significant implications for interpreting microstructural signatures in naturally metasomatized rocks that are common in a variety of crustal and mantle settings^17^.

## Results

### The replacement of KBr by KCl

We discuss 8 representative mineral replacement experiments, during which ∼3 × 4 × 7 mm large single crystals of KBr were partly replaced by a KCl-KBr product phase during exposure to a saturated KCl-H_2_O solution. In the experiments two main parameters were varied (Table 1), the microstructure of the parent KBr crystal (undeformed for set I experiments or crystal-plastically pre-deformed for set II experiments) and the duration of the experiment. The reactions are rapid; the longest experiments of 8 h duration produce >1.5 mm wide replacement rims (Table 1).

**Figure 1:**
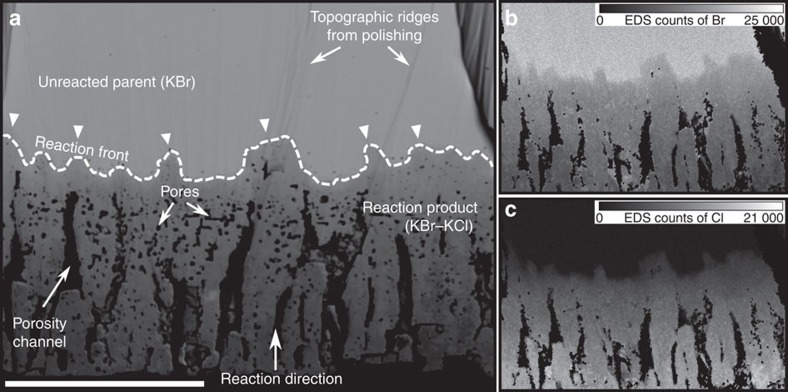
General characteristics of a sample crosscut after experiment. Scale bar, 500 μm. (**a**) Backscatter electron (BSE) micrograph; note the irregularity of the reaction front (white triangles mark highly advanced parts). Some topographic ridges produced by the polishing method can be seen on the image. They are not visible in chemical (**b**,**c**) or crystallographic (c.f., Fig. 3a) maps. (**b**,**c**) EDS counts of Br and Cl in (**a**).

The sample dimensions before and after experiments remain identical, but the replaced part of the samples consist of a solid solution between KBr and KCl and exhibits ∼30% porosity as shown in a cross-section of a partially replaced sample (Fig. 1a–c). The individual pores have sizes of ∼10–50 μm on average, but they can be connected in elongated channels that are up to 500 μm long and oriented normal to the reaction interface. The reaction interface between KBr parent crystal and KBr-KCl products is in general subparallel to the outer surface of the sample (Fig. 1a–c). However, especially in the short experiments μm-scale irregularities in the advancement of the reaction interface can be observed (Fig. 1).

### Crystallographic characteristics of bulk samples

**Figure 2:**
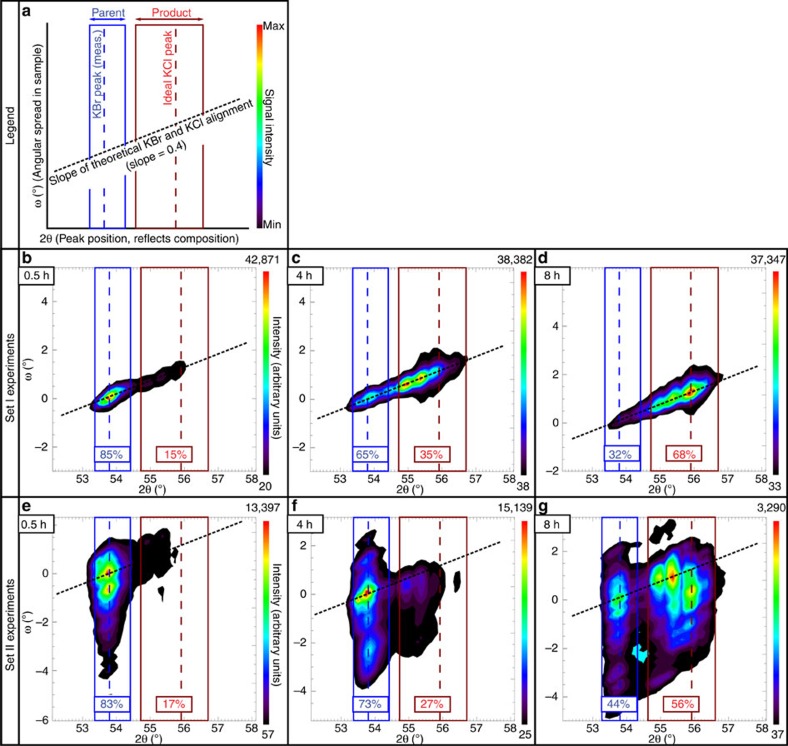
Neutron diffraction data. (**a**) A legend showing the main features noted in the plots. (**b**–**d**) Set I samples. (**e**–**g**) Set II samples. The *x* axis (2θ) represents lattice parameters in the analysed samples, the ideal positions for KBr and KCl are marked with dashed vertical lines and the observed range for parent (KBr) and product (KBr-KCl) reflections are marked with blue and red boxes. The *y* axis (sample ω) represents angular spread (°) in the samples relative to the parent reflection peak. The ω–2θ gradient describes positions where parents and products are in an ideal crystallographic alignment. The mode% of the parents and products are measured and calculated from BSE images of the cross-sections of reacted samples before neutron diffraction analysis.

The *in situ* neutron diffraction analyses allow assessment of the overall, bulk crystallographic orientation of the reacted samples. Results show that with increasing experiment duration the Bragg reflection intensity of the parent's [200] peak (typically at ∼53.8° for KBr) decreases successively, whereas the intensity of product's [200] peak increases and trends towards the ideal KCl [200] reflection at 55.9° (Fig. 2b–d), reflecting the successive change in chemistry with the growth of replacement rims (Fig. 2a). For the undeformed samples, the crystallographic [200] planes of the parent and product phases are perfectly aligned as seen from the alignment of the highest intensities of parent and product peaks along the ω–2θ gradient (Fig. 2b–d). The angular spread of the [200] peak in the undeformed parent phases is low (<1°), whereas the replacing product phases display an increase of the angular spread from ∼0.5° in 0.5 h experiments where only 15% of the sample is replaced to ∼2° in 8 h experiments where 68% of the sample is replaced. For set II experiments in which deformed parent samples are reacted, the highest intensities of the parent and product [200] peaks are again aligned along the theoretical ω–2θ gradient (Fig. 2e–g). The parents show angular spread in a range between 6 and 10° reflecting internal variations in the lattice orientation, whereas the products have smaller spread in the short experiments (Fig. 2b,e), but with increasing reaction times obtain a similar spread to their parents (Fig. 2c,d,f,g). It should be noted that the small spread in the short-duration experiments is expected as only a small part of the pre-deformed crystal has been replaced. It is noticeable, however, that the angular spread even in the short-duration experiments is more than double of that for the set I experiment in which an undeformed parent sample is reacted (Fig. 2b,e).

### Detailed analysis of initial microstructures

**Figure 3:**
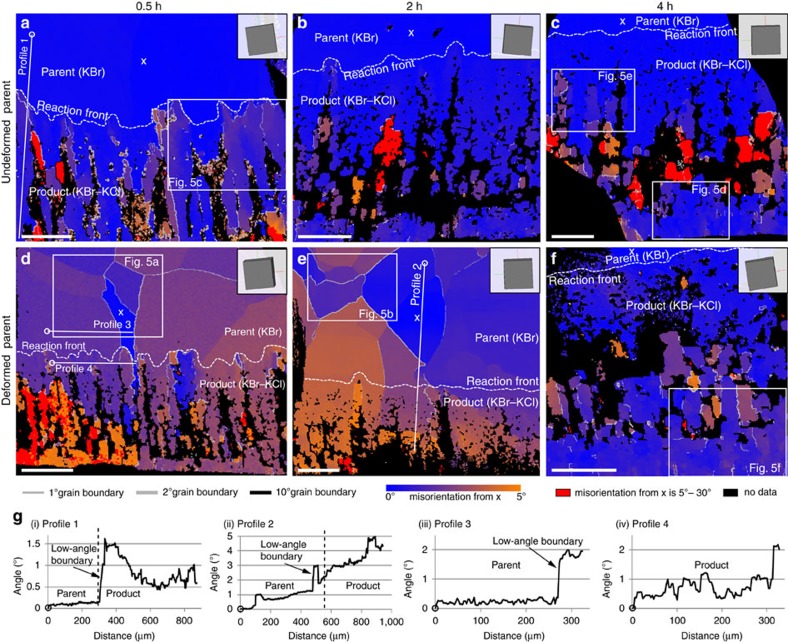
**Electron backscatter diffraction (EBSD) data**. The scale bar on all images is 200 μm. Crystallographic misorientations in sample cross-sections from experiments with undeformed parent samples (set I) (**a**–**c**) and deformed parent samples (set II) (**d**–**f**) relative to the reference point x. The insets on the upper right of each EBSD map shows the physical orientation of the KBr crystal at the reference point x. (**g**) Misorientation angle variations across the profiles marked in (**a**), (**d**), (**e**). Starting point of each profile is marked with (o).

**Figure 4:**
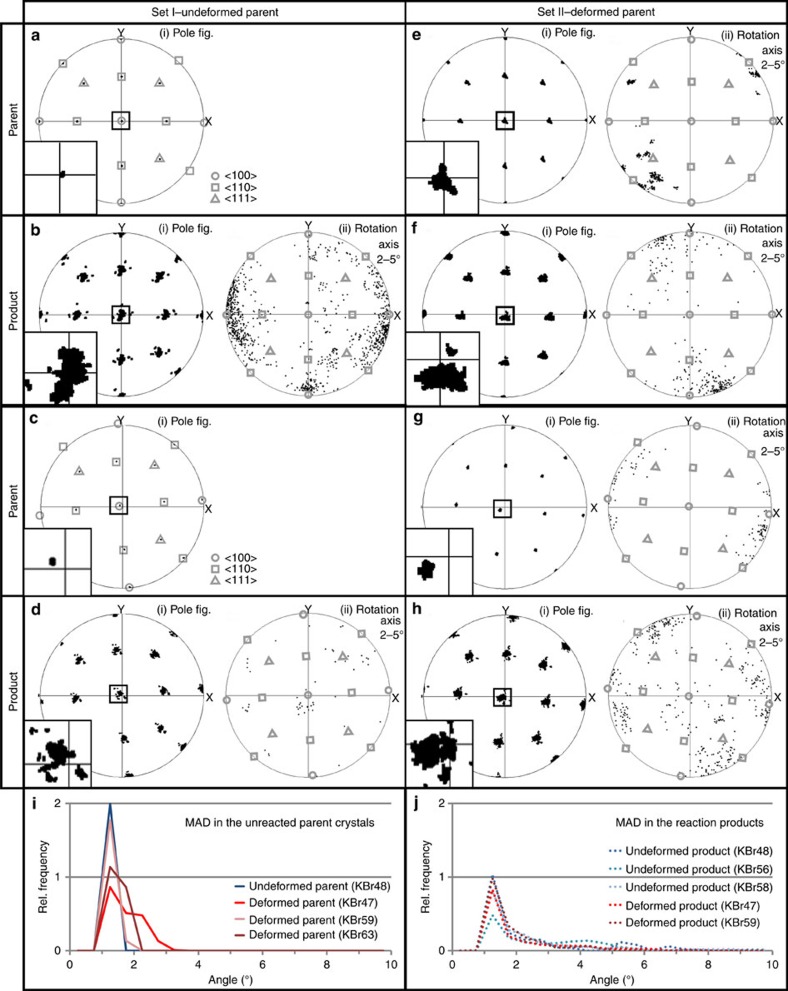
Crystallographic orientations in parents and products. (**a**–**h**) Pole figures and rotation axes for unreacted parts (parents) and reacted parts (products) in samples KBr 56, 49, 48 and 47. (**i**,**j**) Relative frequencies of correlated misorientation angle distribution (MAD) for unreacted (**i**) and reacted parts (**j**) of the experimental samples.

The undeformed parents, used in set I experiments, display minimal internal lattice distortion, mostly within a range of the analytical error (0.2°), low-angle boundaries above 1° are absent (Fig. 3a–c,g(i)), the orientation spread in pole figures is tightly clustered (Fig. 4a,c) and the relative misorientation angle distribution (MAD) has maximum values in a range below 1.25° (Fig. 4i).

**Figure 5:**
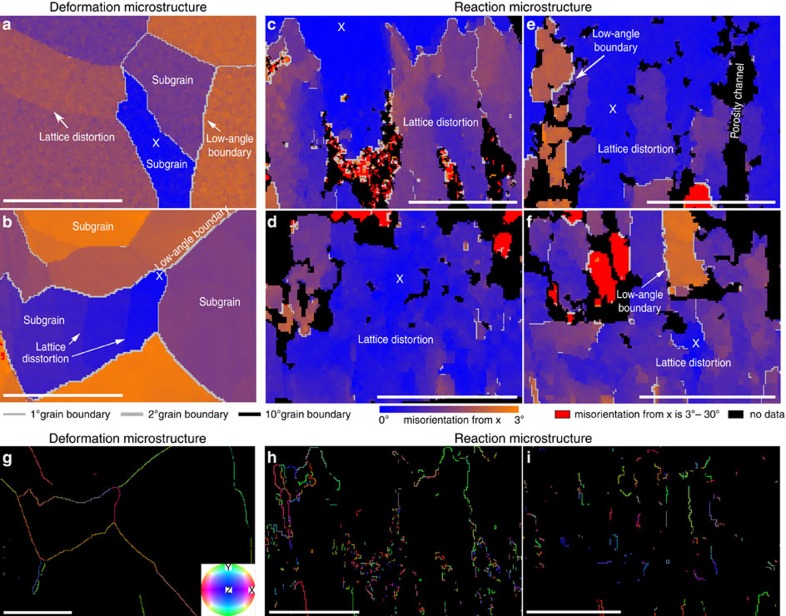
Higher-magnification images of deformation and reaction microstructures. The scale bar on all images is 200 μm. (**a**,**b**) Deformation microstructure in Fig. 3d,e, respectively. (**c**–**f**) Reaction-generated microstructure in Fig. 3a,c,f. Note that the colour scale represents smaller range in misorientation angles compared with Fig. 3 in order to better show structural heterogeneity. (**g**–**i**) Misorientation axes of low-angle boundaries (>1°).

The deformed parents that were used for set II experiments have a polygonal substructure, where distinct, often straight or smoothly curved low-angle boundaries of 1°–2° separate triangular and four-sided subgrain domains (Figs 3d,e,g(ii) and 5a,b,g). These subgrain boundaries are generally parallel to low-index crystallographic planes of the parent grains (Fig. 3a–f and Supplementary Fig. 1) and display consistent rotation axis along individual subgrain segments (Fig. 5g), similar to the microstructures reported in deformed rock salt (NaCl) by Borthwick and Piazolo^18^. Internal deformation is characterized by series of stair-step misorientation profiles where each subgrain is represented by flat or gradually sloping line that fluctuate within a range of 0.2° (Fig. 3g(ii)). The pole figures for the deformed parents in set II experiments show tight clustering of misorientation axes that in some cases branches out in two directions with ∼120° angle between them (Fig. 4e(i)). The 2–5° rotation axes show clustering around [100] direction for sample no. RKBr47 (Fig. 4g(ii)) and several clusters close to [110] direction for sample no. RKBr49 (Fig. 4e(ii)). The MAD is elevated for the angles below 3° but stay close to zero in interval from 3° to 10° (Fig. 4i).

### Detailed analysis of reaction microstructures

The reaction products of all experiments exhibit higher lattice distortion than their parents, but mostly are oriented within a 5° range from the orientation of their adjacent parent grains (Fig. 3a–f). Different to set II parents, the microstructure of the reacted parts of all samples is characterized by elongated domains that are separated by low-angle boundaries of 1–10°. The orientation of these boundaries is generally perpendicular to the reaction interface, and not the crystallographic planes in the parent (Fig. 3d,e). The crystal lattice inside these subgrain domains are more distorted compared with the subgrain interiors in the deformed samples; the variations in lattice misorientation angles in the reaction microstructure are highly irregular and patchy, in contrast to the homogenous, often gradually bent subgrain interiors in the deformation microstructure (Fig. 5a–f). This irregularity is well reflected by the misorientation profiles, were reaction products display higher variation in the misorientation angles across the individual subgrains than the variation in misorientation angles across the lattices in the parent grains (Fig. 3g). The subgrain boundaries in the reaction products are often discontinuous, <50 μm in length and highly irregular; hence distinctly different to the subgrain boundaries in the deformation microstructure (Figs 3a–f and 5). The wide angular range of rotation axes along the same boundary segment in the reaction microstructure is in stark contrast to the distinct rotation axes along each subgrain boundary segment in the deformation microstructure (Fig. 5g–i and Supplementary Fig. 1). All these crystallographic features are even common in reaction products that replace undeformed parent samples. However, the product phases replacing deformed parent crystals often also display continuation of low-angle boundaries from the adjacent parent grains (Fig. 3d,e).

Pole figures of the reaction products also demonstrate a wider spread of misorientation axes (Fig. 4b(i) and higher scattering of 2–5° rotation axes (Fig. 4b(ii)). Interestingly the 2–5° rotation axes in the reaction products do not coincide with [100], [110] or [111] directions and do not resemble the distribution of the rotation axes of the adjacent parent grains (Fig. 4e(ii)). The relative distribution of MAD reaches the maximum values at 1–2° angles, but also is elevated for 2°–7° misorientation angles (Fig. 4j).

## Discussion

All conducted experiments demonstrate a transference of the crystallographic information from the parent minerals into the reaction products, as indicated by the close alignment of parent and product reflection peaks in neutron diffraction data (Fig. 2), and the general coincidence of orientations shown in EBSD maps (Figs 3 and 5) and pole figures (Fig. 4a–h). Although such relationship can be expected in lattice diffusion-controlled reactions, where the general crystallographic framework of parent minerals can be preserved if some of its components are in equilibrium with the system conditions, a number of previous studies^19^,^20^,^21^ have demonstrated that fluid-mediated mineral reactions in KBr-KCl-H_2_O system are not controlled by lattice diffusion, but instead proceed by dissolution–precipitation mechanisms. As shown by Putnis and Mezger^19^, during the chemical equilibration by fluid-mediated dissolution–precipitation processes, the crystallographic framework of the parent mineral is entirely destroyed by the interaction with the fluid. Thus, all microstructures exhibited by products of such reactions are newly generated during the process of the reaction. In this context the coincidence of parent-product crystallographic orientations that is occasionally reported from other hydrothermal studies^19^,^22^,^23^ is interpreted to be a nucleation and growth feature. Furthermore, it has been proposed that such relationship is only possible in systems where parent and product minerals are crystallographically similar^20^ and the dissolution–precipitation processes are kinetically coupled at the reaction front^23^.

Here, we demonstrate that the near-perfect epitaxy during fluid-mediated dissolution–precipitation reactions can occur not only at the overall grain scale, but also to some extent on the intragrain scale. Replacement of deformed parents (set II experiments) creates products that roughly inherit low-angle boundaries, subgrains and subgrain-scale lattice distortions from their adjacent parent grains (Fig. 3d,e). Further on, in addition, microstructures, such as low-angle boundaries, new subgrains and internally distorted crystal lattices (Figs 3a–f and 5c–f) can form directly during the mineral replacement by dissolution–precipitation reactions in the absence of differential stress, and bear striking similarities to the microstructures that form by crystal-plastic deformation in dislocation creep regime (Figs 3d,e and 5a,b). This observation highlights a challenging problem for interpreting metasomatic mineral assemblages, common in many crustal high-strain zones and some mantle settings^17^. Even if the fluid alteration is recognized in the rock, the questions remain: When and how were the microstructures in the metasomatic reaction products created? Did they form directly by crystal-plastic deformation after the metasomatic event? Were they epitaxially inherited during fluid-mediated reactions from previously deformed parent minerals? Or were they entirely generated by fluid-mediated dissolution–precipitation reactions in the absence of deformation? The interpretation of such microstructures will significantly affect the reconstruction of the metasomatic/metamorphic and tectonic events in the rock history.

We suggest that with careful microstructural characterization it is possible to distinguish reaction-produced distortions in the mineral lattices from those that are created by deformation. The key lies in the recognition that even with similar final appearance, the generation of the two microstructures is controlled by different mechanisms. The characteristics of deformation microstructures that form by crystal plasticity are dependent on the crystallographic properties of the mineral, activated slip systems, relative orientation of the deformed grains, main axes of differential stress and extrinsic conditions at the time of deformation^24^,^25^. In contrast, microstructures produced by the reaction depend on the orientation and kinetics of the moving reaction front. Based on the features observed in the experimental samples (Figs 3 and Supplementary Fig. 1) we suggest that the following criteria could be indicative for reaction-generated lattice distortions in minerals, rather than crystal-plastic deformation: (1) highly irregular and discontinuous subgrain and grain boundaries; (2) no correlation between the main crystal lattice orientations and grain boundary orientation; (3) low-angle boundaries and grain boundaries that are dominantly perpendicular to the reaction front; (4) wide range of rotation axes orientations even within the same (sub-)grain boundary segment; (5) irregularly shaped and ‘patchy' subgrains; (6) differing rotation axes distribution and slip system solutions in corresponding parents and reaction products, even when microstructures seem visually similar; and (7) elevated MAD values at larger angles on a subgrain scale.

Although none of these features on its own can be a definite proof that the microstructure is generated by fluid-mediated dissolution–precipitation reactions, in their combination these features are distinct from crystal-plastic deformation-generated microstructures. Such an interpretation will be strengthened if a clear chemical change coincides with the microstructural features outlined. In conclusion, it is important to consider the idea that the presence of subgrain boundaries and seemingly ordered bending of the crystal lattices may not always origin by deformation. Consequently, it is imperative that an in-depth microstructural and microchemical characterization is necessary to allow for reliable interpretation of complex rock histories.

## Methods

### Experimental procedure and sample characterization

All experiments took place at Macquarie University. Two sets of samples were prepared from high purity KBr single crystals (>99% KBr; TedPella, Inc.). Set I samples that represent undeformed single crystals were prepared by cleaving the initial KBr starting material. Set II samples that represent deformed single crystals were prepared by deforming the initial KBr starting material beneath a steel press that was customary made by Macquarie Engineering & Technical Services (METS) (Supplementary Fig. 2) in a high temperature oven, heated to 700 °C for duration of 17 h. No confining pressure was applied. After deformation, samples were cooled down to room temperature over a duration of 12 h. As a result, ∼7–12% shortening was achieved and all set II samples exhibited partly annealed crystal-plastic deformation microstructures, such as subgrains and undulose lattice distortions^5^,^26^ (Fig. 3d,e).

For each reaction experiment, crystals from both sets of samples, the undeformed set I and the deformed set II, were cleaved with a razor blade to sizes of ∼3 × 4 × 7 mm and coated with a water-resistant glue, leaving only one side (4 × 7 mm) exposed for the reactive fluid.

Each crystal was immersed in 15 ml of a saturated KCl-H_2_O solution and kept at room temperature (23±1 °C), and atmosphere pressures for durations from 30 min to 8 h (Table 1). After experiments, samples were removed from the solution and gently dried using a paper tissue.

After experiments samples were characterized by backscatter electron imaging, energy-dispersive X-ray spectroscopy, EBSD and neutron diffraction analysis.

### Chemical properties of KBr-KCl-H_2_O system

The system KBr-KCl-H_2_O was mainly chosen for the experiments in this study because of its well-known chemical properties and solubility data that are easily accessible from a number of previous studies^21^,^27^,^28^,^29^,^30^. The KCl-H_2_O solution that was used as the reactive fluid during the conducted experiments was prepared from 35.1 g KCl on 100 g H_2_O, based on the KCl solubility at 23 °C (Supplementary Fig. 3).

The reaction product KBr-KCl is a solid solution where Cl^−^ substitutes for Br^−^ in the initial KBr crystal lattice. The solubility of this system is characterized by a Lippmann-type phase diagram, where the composition of the KCl product that precipitates during the performed experiments is a function of the solution composition that is continuously modified by the dissolution of the parent KBr crystal^2^. As a result reaction rims show a compositional gradient characterized by decreasing Cl concentration towards the sample interior (Supplementary Fig. 4).

### Crystallographic properties of KBr and KCl

The experimental system involves two minerals, KBr as a parent mineral and KCl as a reaction product that pseudomorphically replaces KBr during the experiments. Both minerals are salts with cubic, face-centred crystal lattices. The crystallographic framework of both phases consist of two interpenetrating cubic, face-centred lattices, one of K and one of Br or Cl. The ions in this structure is arranged in a way that each K cation is octahedrally surrounded by six Br or Cl anions and each Br or Cl anion is in same way surrounded by six K cations. Ionic radius of K^+^=1.38 Å , Br^−^=1.96 Å and Cl^−^=1.81 Å^31^. Crystals cleave easily in three directions parallel to (100).

Supplementary Table 1 provides characteristics of the specific structural solutions that were used for identification of the crystallographic patterns during EBSD mapping.

Supplementary Figure 5 illustrates the physical orientation of planes and directions in cubic crystal systems that were used to plot pole figures and rotation axis in the Fig. 4.

### Sample preparation for EBSD analysis

After the experiments, ∼2 mm thick slices were cleaved from the central parts of each sample, perpendicular to the reacted surface, so the interface between the parent crystal and reaction rim was exposed in the cross-sections. As KBr and KCl are highly soluble in water, the polishing for EBSD analysis was performed with a focussed argon (Ar) ion beam using IB-09010CP Cross Section Polisher at OptoFab node, Australian National Fabrication Facility. Each sample was polished for 2–3 h using a beam with a diameter of 500 μm, ∼190–210 μA top current and ∼80–90 μΑ bottom current (Supplementary Fig. 6).

### EBSD data collection and analysis

Crystallographic orientation data were acquired using Carl Zeiss EVO MA15 scanning electron microscope with HKL NordlysNano high-sensitivity EBSD detector at the Geochemical Analysis Unit in Macquarie University. The EBSD and energy-dispersive spectroscopy analyses were performed simultaneously with AzTec analysis software (Oxford Instruments) on samples tilted to 70° angle at high-vacuum conditions with accelerating voltage of 20–30 kV and beam current of 8–8.2 nA at working distances from 17 to 22 mm. The high working distances were used to avoid shadowing effects from edges surrounding the ion beam polished area. Step sizes of 3 μm were used for all maps.

Crystallographic orientation data were processed using Channel 5 analysis software from HKL Technology. Noise reduction included the removal of wild spikes, extrapolation of zero solutions by iteration to 6 neighbours and application of Kuwahara filter with the grid size of 3 × 3, smoothing angle of 2° and artefact angle of 1°.

For the presented EBSD maps, the KBr-KCl reaction rim was always indexed using KBr structural solution that showed a better fit than KCl unit cell. To test whether the choice of the indexed phase does not affect the obtained orientation data, raw Kikuchi patterns were reanalysed using KCl structural solution. As shown in Supplementary Fig. 7, no significant effect was detectable, but the range of orientation and the amount of subgrain boundaries (especially in Supplementary Fig. 7a) is higher than if indexed with KBr solution (Fig. 3a,e).

Slip systems for individual subgrain boundaries were determined following the procedures of Prior *et al*.^32^, Reddy *et al*.^33^ and Piazolo and Jaconelli^34^. Supplementary Figure 8 shows the typical slip system solutions for subgrain boundaries in the product phase from set I experiments (Supplementary Fig. 8a) and parent (Supplementary Fig. 8b) and product (Supplementary Fig. 8c) phases from set II experiments. The analysed subgrain boundaries are marked on Supplementary Fig. 8d,e.

Supplementary Figure 1 show the low-angle and grain boundary orientations for all samples depicted in Fig. 3.

### Neutron diffraction analysis

Bulk crystallographic properties of the experimental samples were characterized using high-intensity neutron powder diffractometer (WOMBAT) at the Australian Centre for Neutron Scattering in the Australian Nuclear Science and Technology Organisation. Analysis was performed on ∼4 mm thick slices cleaved from 6 partly replaced samples (RKBr 56, 57, 58, 59, 60 and 61; Table 1). The neutron diffraction data were collected in 0.2° steps for the [200] peaks using incident wavelength of 2.95 Å with no radial collimator movement. The diffraction intensities were plotted in two-dimensional graphs (Fig. 2) for each analysed sample, where *x* axis represents sample lattice parameters, measured as 2θ angles, and *y* axis represents angular spread in the samples, measured as sample ω angles. The peak position for KBr [200] on 2θ (plotted as blue dashed line in Fig. 2) is consistent in all samples and was estimated from the maximum reflection intensity to be ∼53.8° that equates to a lattice parameter of a −6.52 Å. As product phase in all experiments is a solid solution between KBr and KCl, the peak position for KCl [200] could not be estimated from the measured data. Instead, a measurement of Slagle and McKinstry^35^ at 55.9° was used as a standard (red dashed line in Fig. 2).

The percentages of the parent and product that are plotted on Fig. 2 were estimated by measuring and calculating the two-dimensional areas of the reaction rims and parents on backscatter electron images of the samples that were subsequently analysed by Neutron diffraction.

### Data availability

The data and material used for this study are contained within the article, Supplementary Information or available from the corresponding author L.S. on request.

## Additional information

**How to cite this article:** Spruzeniece, L. *et al*. Deformation-resembling microstructure created by fluid-mediated dissolution-precipitation reactions. *Nat. Commun.* **8,** 14032 doi: 10.1038/ncomms14032 (2017).

**Publisher's note:** Springer Nature remains neutral with regard to jurisdictional claims in published maps and institutional affiliations.

## Supplementary Material

Supplementary Information Supplementary Figures, Supplementary Table and Supplementary Reference.

## Figures and Tables

**Table 1:** Samples and experimental conditions.

**Conditions: T=22–23 °C; P=atmosphere; fluid=saturated KCl–H**_**2**_**O**					
**Exp. no.**	**Exp. set**	**Exp. duration (h)**	**Strain in parent (% of shortening)**	**Reaction rim (μm)**	**Replacement (%)**
RKBr56*	I	0.5	0	516	15
RKBr57*	II	0.5	11	540	17
RKBr48	I	2	0	700	24
RKBr49	II	2	12	620	22
RKBr58*	I	4	0	1,020	35
RKBr59*	II	4	7	900	27
RKBr60*	I	8	0	1,650	68
RKBr61*	II	8	13	1,630	56

Exp., experiment.

Samples from experiments that are marked with asterisk were used for characterization with neutron diffraction analysis (Fig. 2).
