# Supplementary material for: Deformation-resembling microstructure created by fluid-mediated dissolution–precipitation reactions
Source: Nat Commun. 2017 Jan 27;8:14032. doi: 10.1038/ncomms14032 (PMC5290167; doi:10.1038/ncomms14032)
Supplement: Supplementary Information — Supplementary Figures, Supplementary Table and Supplementary Reference. [file ncomms14032-s1.pdf]

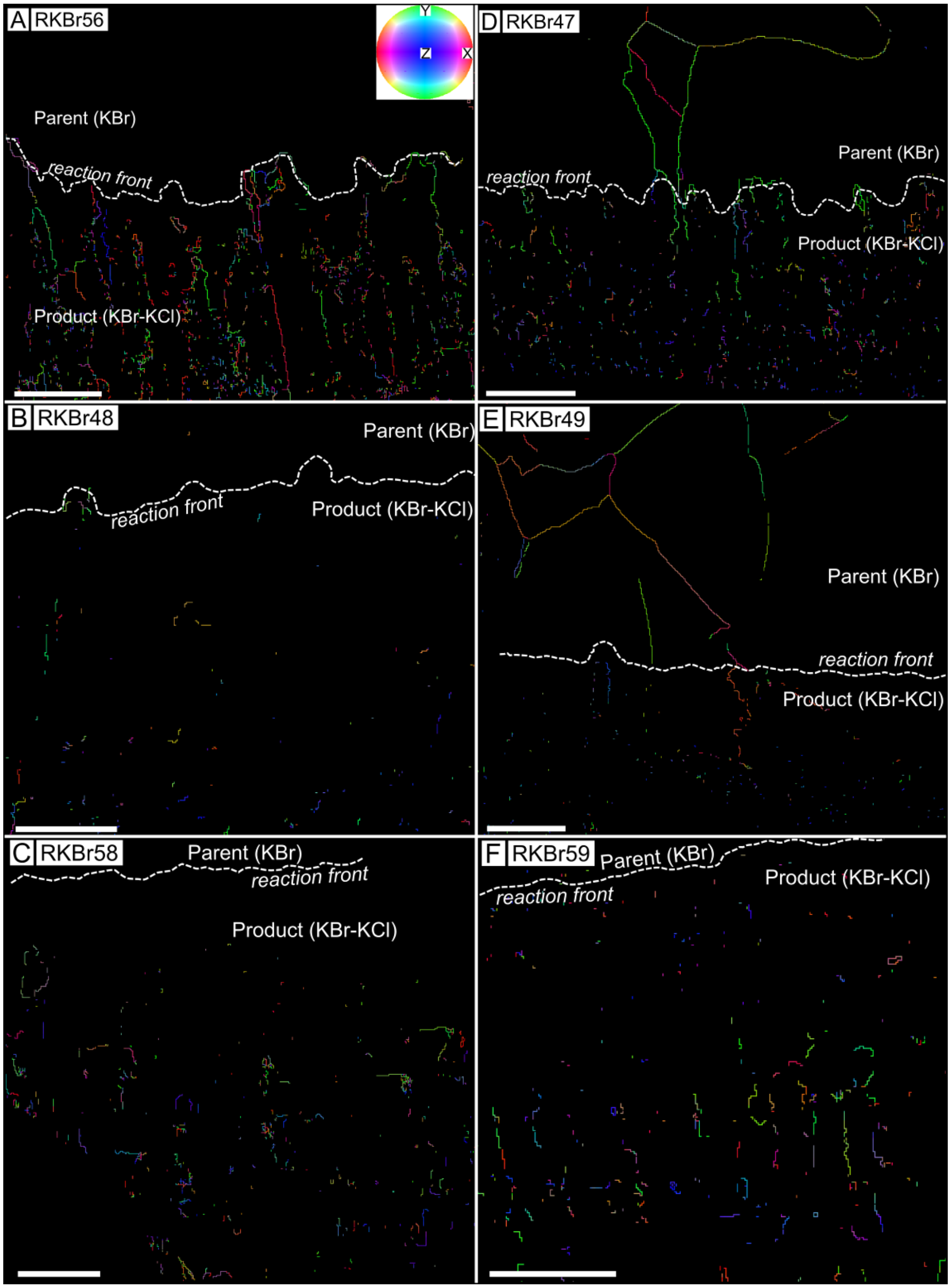

Supplementary Figure 1. Color coded misorientation axes of low angle boundaries (>1°) in the representative experimental samples. (a)-(c) Samples from set I experiments with undeformed KBr parent crystals. (d)-(e) Samples from set II experiments with deformed KBr parent crystals.

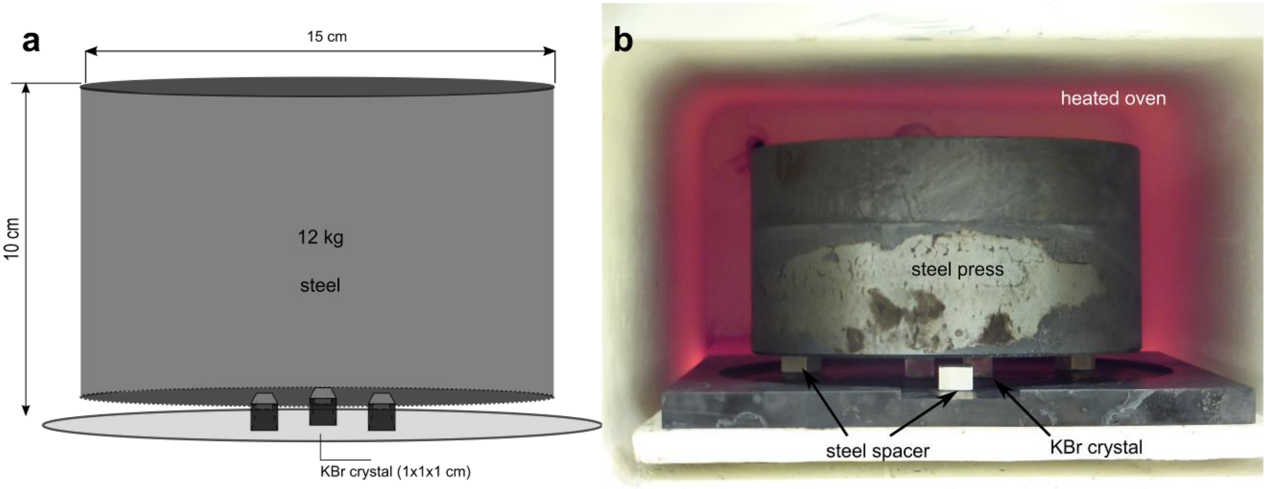

Supplementary Figure 2. Setup for KBr deformation experiments. (a) The design of the steel press. (b) The photo of an experiment in progress.

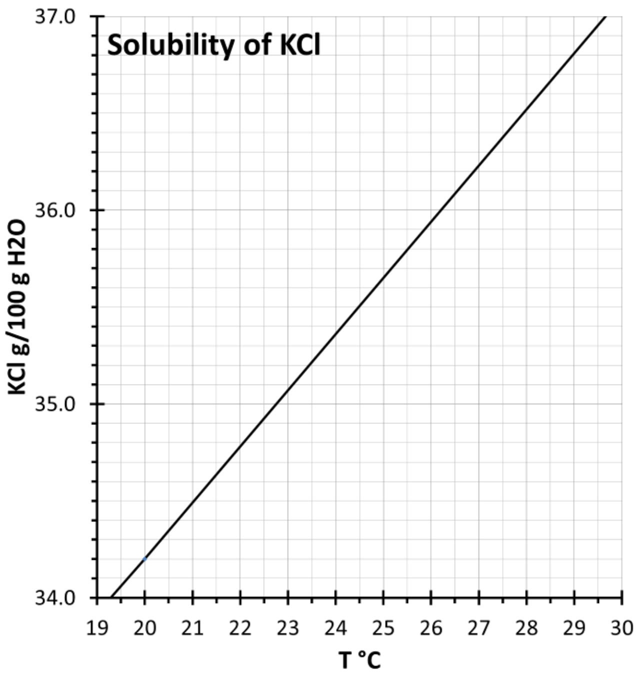

Supplementary Figure 3. KCl solubility in 100 g of H_2O. Based on the data from McGlashan[1]

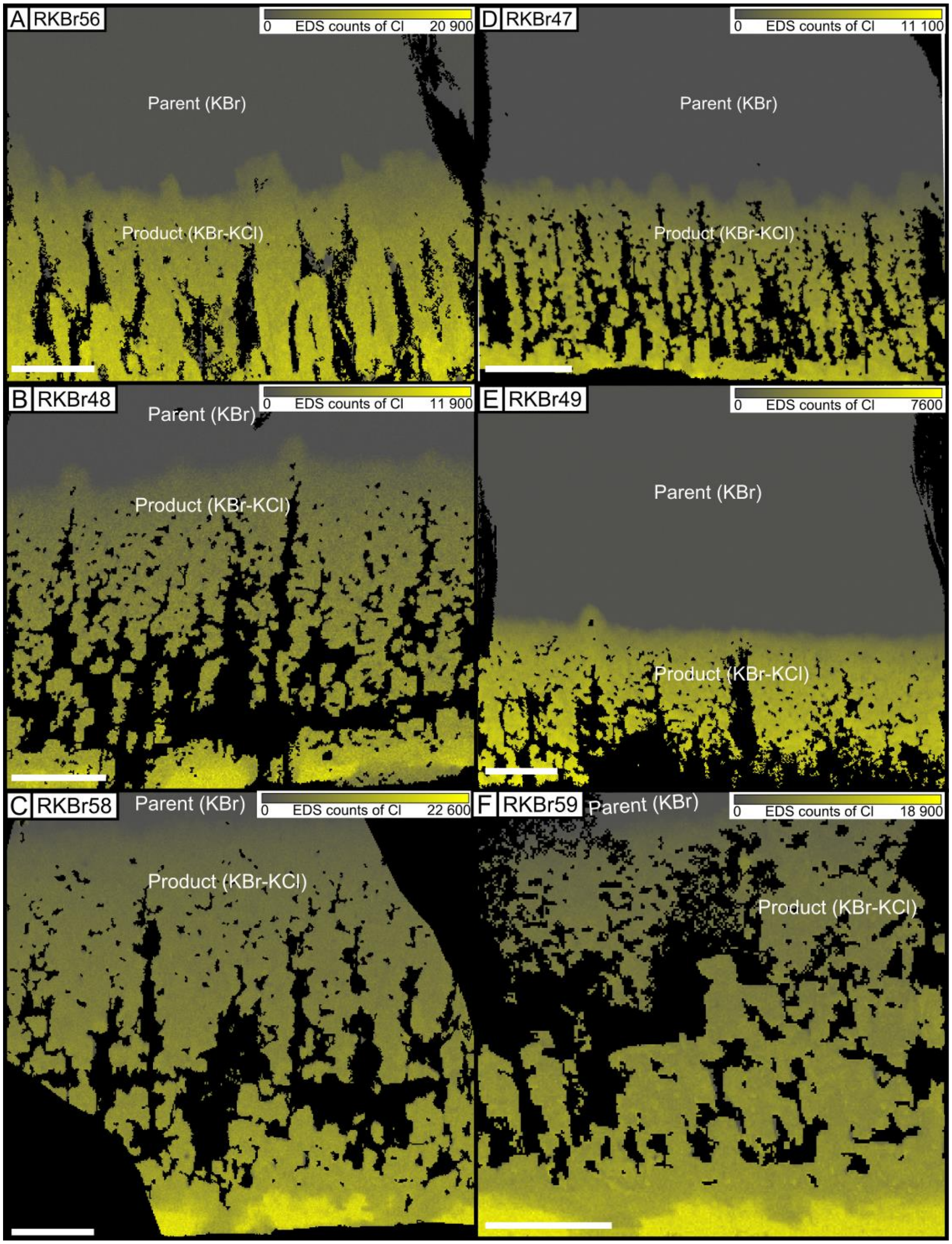

Supplementary Figure 4. EDS maps of Cl distribution in the samples after experiments. The parent crystal is characterized by the absence of Cl, while the product contains various amounts of Cl. The boundary of Cl disappearance in these maps were used to define the reaction front in Fig. 3. (a)-(c) Samples from set I experiments with undeformed KBr parent crystals. (d)-(e) Samples from set II experiments with deformed KBr parent crystals.

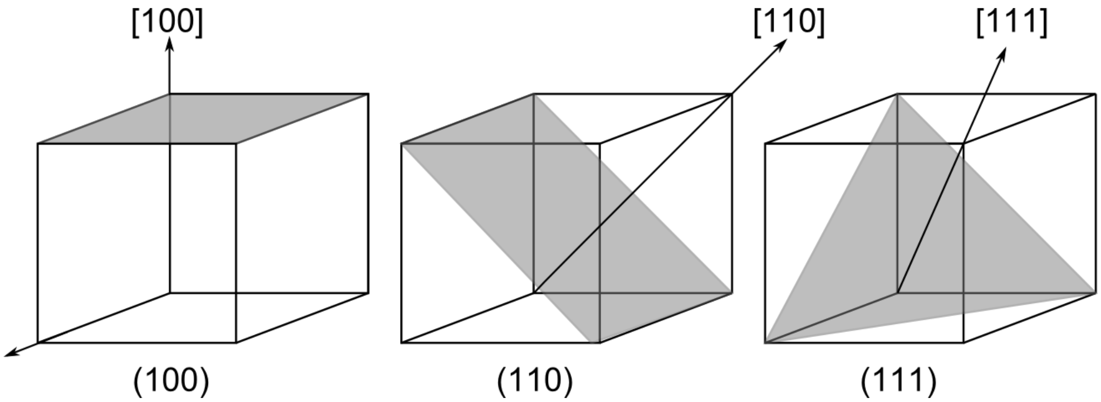

Supplementary Figure 5. Planes and directions in cubic crystal systems. Round brackets and shaded areas depict the planes, while square brackets and arrows depict directions.

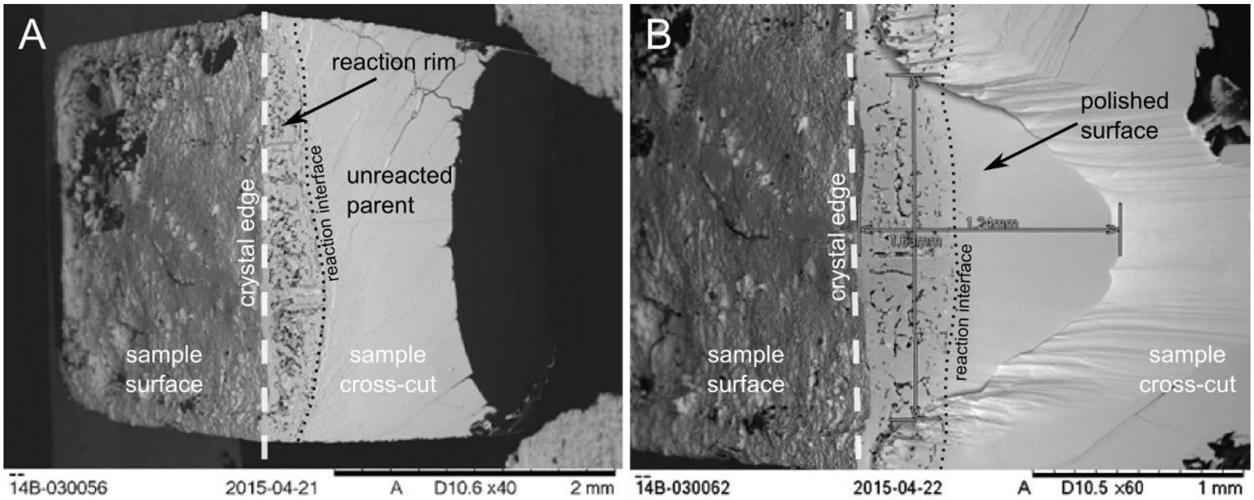

Supplementary Figure 6. Reacted sample before (a) and after (b) ion beam polishing. The image shows two crystal faces of the sample as it is mounted on a tilted SEM stage (separated by the white dashed line). The rough surface on the left from the crystal edge is the reacted outside surface that was in contact with the reaction fluid. The lighter surface on the right is a cross-cut of the sample after experiment revealing the interface between the reaction rim and unreacted parent grain (marked with black dotted line). (b) A polished surface with dimensions 1.24x1.63 μm in the central part of the cross-cut side of the sample.

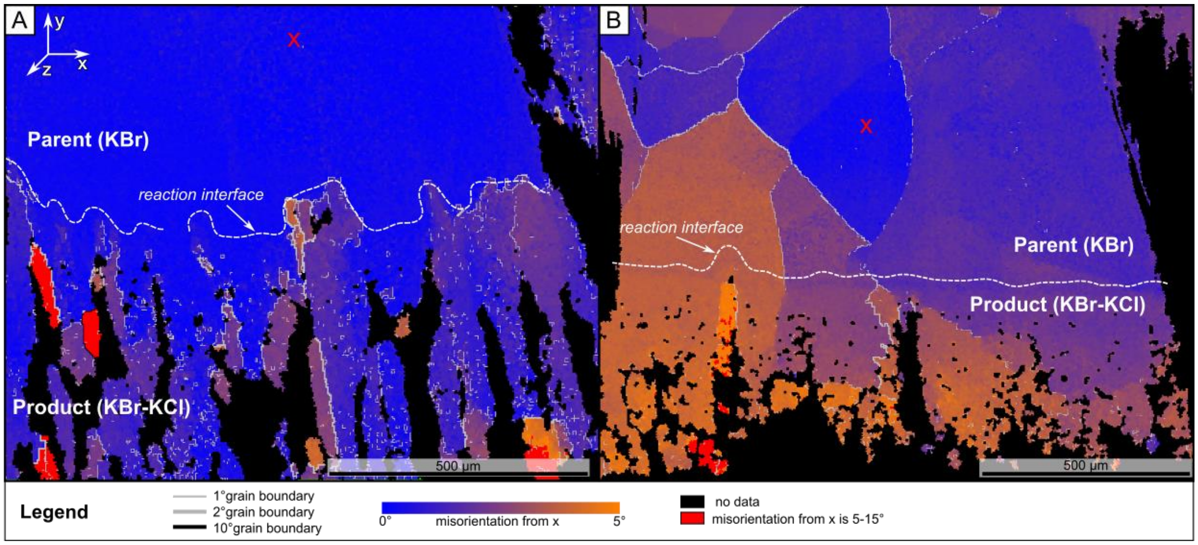

Supplementary Figure 7. Samples depicted in Fig.3a & d reanalyzed using KCl structural solution. (a) Undeformed, partly replaced sample. (b) Deformed partly replaced sample. Note that although the graininess and the amount of low angle boundaries is higher, the general microstructure is very similar as in Fig. 3a & d.

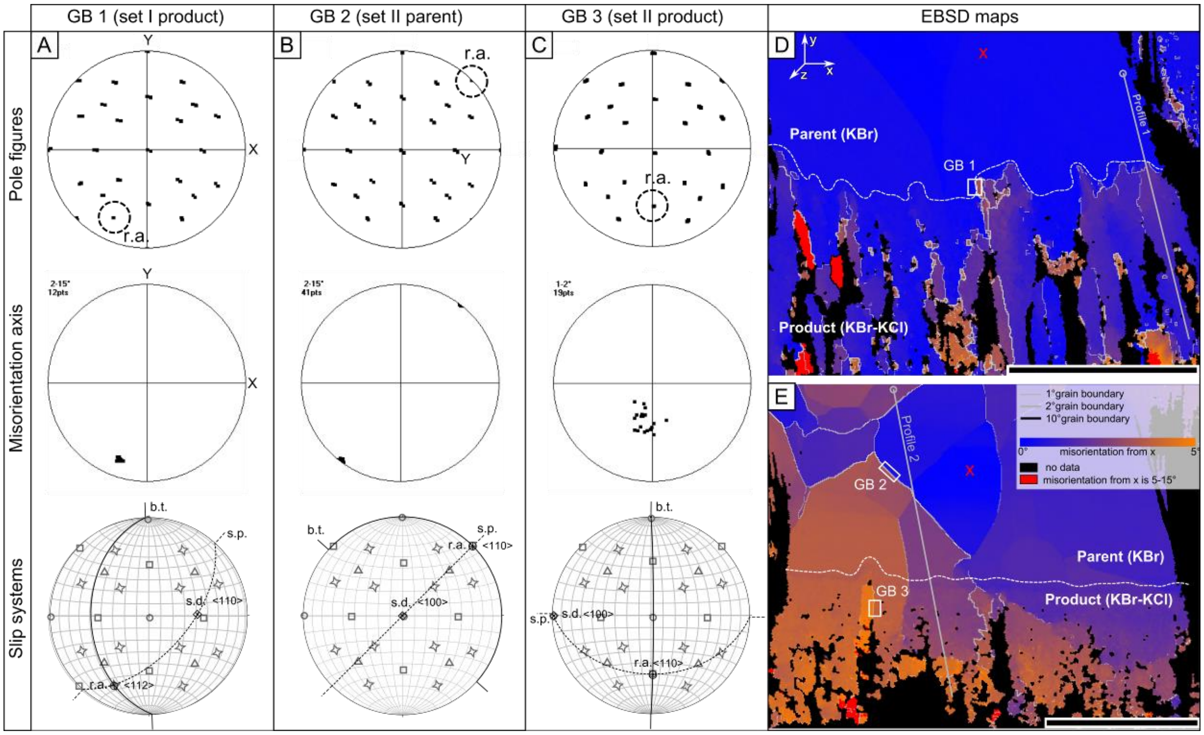

Supplementary Figure 8. Subgrain boundary trace analysis. The analysed grain boundaries (GB) in (a)-(c) are marked in figures (d) and (e). Slip system solutions typically show tilt boundary geometries, however for GB 2, twist boundary is also possible, although not showed in the figure.

Supplementary Table 1. Crystallographic properties of KBr and KCl structures that were matched to Kikutchi bands.

**Mineral**	**KBr**	**KCl**
Crystal system	Cubic – high	Cubic – high
Laue Group	11 (m-3m)	11 (m-3m)
Space group	225 (F m-3 m)	221 (P m -3 m)
Unit cell a (Å)	6.6000	3.6344
Unit cell b (Å)	6.6000	3.6344
Unit cell c (Å)	6.6000	3.6344
Composition	K=50%, Br=50%	K=50%, Cl=50%

SUPPLEMENTARY REFERENCES

1. McGlashan, M.L. Solubilities of solids in water. *http://www.kayelaby.npl.co.uk/chemistry/3_6/3_6_2.html*. (2015).
